# Death registration coverage 2019–2021, India

**DOI:** 10.2471/BLT.22.288889

**Published:** 2022-11-25

**Authors:** Nandita Saikia, Krishna Kumar, Bhaswati Das

**Affiliations:** a Department of Public Health and Mortality Studies, International Institute for Population Sciences, Mumbai, India.; b School of Social Sciences, Jawaharlal Nehru University, New Mehrauli Road, New Delhi-110067, India.

## Abstract

**Objective:**

To investigate coverage and factors associated with death registration in India.

**Methods:**

We used data from the Indian National Family Health Survey 2019–2021. Based on responses of eligible household members, we estimated death registration in 84 390 deaths in all age groups across the country. We used multilevel logistic regression analysis to determine sociodemographic variables associated with death registration at state, district and individual levels.

**Findings:**

Nationally, 70.8% (59 748/84 390) of deaths were registered. Of 707 districts in our study period, 122 and 53 districts had death registration levels less than 40% in females and males, respectively. The likelihood of death registration was significantly lower for females than males (adjusted odds ratios, aOR: 0.61; 95% confidence interval, CI: 0.59–0.64). Death registration increased significantly with age of the deceased person, with the highest odds in 35–49-year-olds (aOR: 5.05; 95% CI: 4.58–5.57) compared with 0–4-year-olds. Death registration was less likely among rural households, disadvantaged castes, the poorest wealth quintile, Muslims and households without a below poverty level card. Higher education was associated with higher death registration with the greatest likelihood of registration in households with a member with post-secondary school education (aOR: 1.54; 95% CI: 1.42–1.66). District-level factors were not significantly associated with death registration.

**Conclusion:**

Sociodemographic characteristics of the deceased person were significantly associated with death registration. Strategies to raise awareness of death registration procedures among disadvantaged population groups and the introduction of a mobile telephone application for death registration are recommended to improve death registration in India.

## Résumé

**Objectif:**

Étudier la couverture et les facteurs associés au système d'enregistrement des décès en Inde.

**Méthodes:**

Nous avons utilisé les données issues de l'Enquête nationale sur la santé des familles réalisée en Inde entre 2019 et 2021. À la lumière des réponses fournies par les membres des ménages pris en compte, nous avons estimé que 84 390 décès étaient enregistrés dans toutes les catégories d'âge à travers le pays. Nous avons ensuite procédé à une analyse de régression logistique multiniveaux afin de déterminer les variables sociodémographiques liées à l'enregistrement des décès à l'échelle des États, des districts et des individus.

**Résultat:**

Sur le plan national, 70,8% (59 748/84 390) des décès ont été consignés. Sur un total de 707 districts repris dans la période couverte par notre étude, 122 affichaient un taux d'enregistrement inférieur à 40% pour les femmes, tandis que 53 affichaient un taux d'enregistrement inférieur à 40% pour les hommes. La probabilité d'enregistrement des décès était nettement moins élevée pour les femmes que pour les hommes (odds ratio ajusté, ORA: 0,61; intervalle de confiance de 95%, IC: 0,59–0,64). L'enregistrement des décès augmentait considérablement avec l'âge de la personne concernée, la probabilité la plus élevée s'observant chez les 35–49 ans (ORA: 5,05; IC de 95%: 4,58–5,57), à l'inverse des 0–4 ans. Les décès étaient moins susceptibles d'être consignés dans les milieux ruraux, les castes défavorisées, les quintiles de richesse les plus bas, les musulmans et les ménages ne possédant pas de carte BPL (octroyée à ceux vivant sous le seuil de pauvreté). Un niveau d'éducation plus élevé allait généralement de pair avec un meilleur taux d'enregistrement des décès; les ménages avec un membre ayant suivi un enseignement supérieur présentaient la plus forte probabilité de recensement (ORA: 1,54; IC de 95%: 1,42–1,66). Les facteurs à l'échelle des districts n'ont pas eu d'impact significatif sur l'enregistrement des décès.

**Conclusion:**

L'enregistrement des décès s'est avéré étroitement lié aux caractéristiques sociodémographiques des personnes décédées. Nous recommandons le déploiement de stratégies visant à mieux faire connaître les procédures de recensement au sein des populations défavorisées, et à introduire une application sur téléphone mobile afin d'améliorer l'enregistrement des décès en Inde.

## Resumen

**Objetivo:**

Investigar la cobertura y los factores asociados al registro de defunciones en la India.

**Métodos:**

Se utilizaron datos de la Encuesta Nacional de Salud Familiar de la India de 2019 a 2021. Según las respuestas de los miembros del hogar que cumplían los requisitos, se estimó el registro de defunciones en 84 390 muertes en todos los grupos de edad de todo el país. Se aplicó un análisis de regresión logística multinivel para determinar las variables sociodemográficas asociadas al registro de defunciones a nivel estatal, distrital e individual.

**Resultados:**

A nivel nacional, se registró el 70,8 % (59 748/84 390) de las muertes. De los 707 distritos incluidos en el periodo de estudio, 122 y 53 distritos tenían niveles de registro de defunciones inferiores al 40 % en mujeres y hombres, respectivamente. La probabilidad de registro de defunciones fue significativamente inferior en el caso de las mujeres que en el de los hombres (razones de posibilidades ajustadas, RPA: 0,61; intervalo de confianza del 95 %, IC: 0,59-0,64). El registro de las defunciones aumentó significativamente con la edad de la persona fallecida, con las posibilidades más altas en las personas de 35 a 49 años (RPA: 5,05; IC del 95 %: 4,58-5,57) en comparación con las personas de 0 a 4 años. El registro de las defunciones fue menos probable entre los hogares rurales, las castas desfavorecidas, el quintil de riqueza más pobre, los musulmanes y los hogares sin certificado de pobreza. La educación superior se asoció a un mayor registro de las defunciones, con la mayor probabilidad de registro en los hogares con un familiar que tuviera estudios postsecundarios (RPA: 1,54; IC del 95 %: 1,42-1,66). Los factores a nivel de distrito no se asociaron significativamente con el registro de defunciones.

**Conclusión:**

Las características sociodemográficas de la persona fallecida se asociaron de manera significativa con el registro de defunciones. Se recomiendan estrategias de concienciación sobre los procedimientos de registro de defunciones entre los grupos de población desfavorecidos y la introducción de una aplicación de telefonía móvil para el registro de defunciones con el fin de mejorar el registro de defunciones en la India.

## ملخص

**الغرض:**

التحقيق بشأن تغطية تسجيل الوفيات والعوامل المرتبطة بها في الهند.

**الطريقة:**

قمنا باستخدام بيانات من المسح الوطني الهندي لصحة الأسرة 2019 إلى 2021. بناءً على إجابات أفراد الأسرة المؤهلين، قمنا بتقدير تسجيل الوفيات في عدد 84390 حالة وفاة في كل الفئات العمرية في كافة أنحاء البلاد. قمنا باستخدام تحليل التحوّف اللوجستي متعدد المستويات لتحديد المتغيرات الاجتماعية السكانية المرتبطة بتسجيل الوفيات على مستوى كل من الولاية والمنطقة، وعلى المستوى الفردي.

النتائج على الصعيد الوطني، تم تسجيل 70.8% (59748/84390) من الوفيات. من بين 707 منطقة في فترة الدراسة، كان لدى 122 و53 مقاطعة مستويات تسجيل وفيات أقل من 40% في الإناث والذكور، على التوالي. كان احتمال تسجيل الوفيات أقل بشكل واضح للإناث منه للذكور (نسب الاحتمالات المعدلة: 0.61؛ بفاصل ثقة مقداره 95%: 0.59 إلى 0.64). زاد تسجيل الوفيات بشكل ملحوظ مع تقدم عمر الشخص المتوفى، مع أعلى احتمالات عند الأشخاص الذين تتراوح أعمارهم بين 35 و49 عامًا (نسب الاحتمالات المعدلة: 5.05؛ ‏بفاصل ثقة مقداره 95%: 4.58 إلى 5.57) مقارنةً بالأفراد منذ الولادة إلى عمر 4 سنوات. كان تسجيل الوفيات أقل احتمالاً بين الأسر الريفية، والطبقات المحرومة، والفئة الخمسية الأكثر فقرًا، والمسلمين، والأسر التي ليس لديها بطاقة تحت مستوى الفقر. ارتبط التعليم العالي بتسجيل أعلى للوفيات مع أعلى احتمالية للتسجيل في الأسر التي لديها فرد حاصل على تعليم ما بعد المدرسة الثانوية (نسب الاحتمالات المعدلة: 1.54؛ ‏بفاصل ثقة مقداره 95%: 1.42 إلى 1.66). لم تكن العوامل على مستوى المنطقة مرتبطة بشكل كبير بتسجيل الوفيات.

**الاستنتاج:**

ارتبطت الخصائص الاجتماعية السكانية للشخص المتوفى بشكل كبير بتسجيل الوفاة. يوصى باتباع استراتيجيات لزيادة الوعي بإجراءات تسجيل الوفيات بين الفئات السكانية المحرومة، وطرح تطبيق الهاتف المحمول لتسجيل الوفيات، وذلك لتحسين تسجيل الوفيات في الهند.

## 摘要

目的 旨在调查印度死亡登记覆盖率及相关因素。

**方法:**

我们使用了 2019–2021 年印度全国家庭健康调查中的数据。根据符合条件的家庭成员所提供的答案,我们估计全国所有年龄段的死亡登记人数总计为 84,390 人。我们使用多水平逻辑回归分析方法来确定邦级、县级和个人层面的死亡登记相关社会人口统计学变量。

**结果:**

全国死亡登记率为 70.8% (59,748/84,390)。在我们研究期间,在 707 个县中,分别有 122 个县和 53 个县的女性和男性死亡登记率均低于 40%。女性的死亡登记几率明显低于男性(调整后优势比,aOR:0.61;95% 置信区间,CI:0.59-0.64)。逝者年龄越大,死亡登记几率明显越高,死亡登记率最高的年龄段为 35–49 岁(与 0–4 岁儿童相比,aOR:5.05;95% CI:4.58–5.57)。农村家庭、弱势阶层、五分之一最贫困人群、穆斯林教徒以及未收到定量供应卡的贫困线以下家庭进行死亡登记的可能性较小。高等教育有利于提高死亡登记率,如果家里有人达到了专上教育水平,则该类家庭进行死亡登记的可能性最大 (aOR:1.54;95% CI: 1.42–1.66)。县级因素与死亡登记之间不存在明显相关性。

**结论:**

逝者的社会人口统计学特征与死亡登记明显相关。建议采取策略来提高弱势群体对死亡登记程序的认识,并推出通过手机申请死亡登记的方法,以提高印度的死亡登记率。

## Резюме

**Цель:**

Изучить охват и факторы, связанные с регистрацией случаев смерти в Индии.

**Методы:**

Были использованы данные из Национального обследования состояния здоровья семей в Индии в 2019–2021 гг. На основании ответов соответствующих, совместно проживающих лиц была проведена оценка регистрации смерти в 84 390 случаях во всех возрастных группах по всей стране. Для определения социально-демографических переменных, связанных с регистрацией случаев смерти на уровне штата, района и отдельного человека, использовался многоуровневый логистический регрессионный анализ.

**Результаты:**

На национальном уровне было зарегистрировано 70,8% (59 748/84 390) случаев смерти. За период исследования из 707 районов уровень регистрации случаев смерти среди женщин и мужчин составил менее 40% в 122 и 53 районах соответственно. У женщин вероятность регистрации смерти была значительно ниже, чем у мужчин (скорректированное отношение шансов, сОШ: 0,61; 95%-й ДИ: 0,59–0,64). Показатели регистрации случаев смерти значительно увеличивались с возрастом умершего, причем самые высокие шансы наблюдались в возрасте 35–49 лет (сОШ: 5,05; 95%-й ДИ: 4,58–5,57) по сравнению с детьми в возрасте 0–4 лет. Вероятность регистрации случаев смерти была ниже среди сельских домохозяйств, неблагополучных каст, беднейшего квинтиля по уровню доходов, мусульман и домохозяйств без продовольственных карточек BPL. Высшее образование было связано с более высокой степенью регистрации случаев смерти, причем наибольшая вероятность регистрации отмечалась в домохозяйствах, в которых член домохозяйства имел образование уровня выше среднего (сОШ: 1,54; 95%-й ДИ: 1,42–1,66). Факторы на уровне района не были существенно связаны с регистрацией случаев смерти.

**Вывод:**

Социально-демографические характеристики умершего человека были в значительной степени связаны с регистрацией смерти. Для усовершенствования регистрации случаев смерти в Индии рекомендуется использовать стратегии повышения осведомленности о процедурах регистрации смерти среди малообеспеченных групп населения и разработать специальное приложение для мобильного телефона.

## Introduction

Accurate mortality statistics are indispensable for health-care planning, resource allocation and policy-making.^1^ Mortality data by age, sex, cause of death and place of residence are essential for health officials and decision-makers to identify health threats and high-risk populations.^2^ Sustainable development goal (SDG) 3 aims to “ensure healthy living and promote well-being for all ages by 2030.” The goal primarily includes targets to reduce maternal and child death rates and other premature deaths from noncommunicable diseases.^3^ Measurement of these targets is difficult without complete death registration.^4^ SDG indicator 17.19.2 calls for 100% birth and 80% death registration by 2030 for monitoring statistical capacity.^3^ The World Health Organization SCORE report also suggests strengthening overall health data systems, improving their death registration and collecting better quality data to address inequality.^5^

A civil registration system needs to produce high-quality mortality statistics. Yet, in many low- and middle-income countries, this system is still deficient. The Demographic Health Surveys (DHS) provide high-quality data on child deaths, however, sample sizes of deaths in adults and elderly people are not enough for robust mortality estimates. In addition, DHS do not provide mortality data for vulnerable populations because of sample size restrictions. Thus, estimation of mortality is challenging in the absence of a civil registration system. The quality of mortality information is unsatisfactory in many low- and middle-income countries because death registration in these countries is not universal.^6^^,^^7^ Notably, most people in Africa and Asia do not register births and deaths, which has implications for legal identity and official statistics.^7^^,^^8^ Accurate death registration data also help the judiciary and individuals to resolve inheritance problems fairly.^2^^,^^9^ The coronavirus disease 2019 (COVID-19) pandemic highlighted the importance of accurate counting of deaths to facilitate monitoring the course of the disease.

In India, the Registration of Births and Deaths Act mandated the registration of all births and deaths within 21 days.^10^ The civil registration system of India is a hierarchical structure with registrars located at the local level, district registrars at the district level, chief registrars at the state level and the Registrar General of India at the national level. Deaths are registered with the registrars who compile reports to be sent to higher levels for the state and national reports on death registration. The office of the Registrar General prepares a comprehensive report every year. The number of registered deaths went up from 7.64 million deaths in 2019 to 8.12 million in 2020 – an increase of 6.3%. As per this report, death registration increased to 92% (7 641 076/8 301 769) in 2019 from 85% (6 950 607/8 212 576) in 2018. Nonetheless, some regions still lack complete death registration.^10^

Mortality data from the civil registration systems in India have not been used much in policy framing and health interventions because of the high levels of underreporting of deaths in many states.^9^ Studies in other countries showed that death registration was higher among educated mothers, main ethnic groups and non-poor households.^8^^,^^11^ However, few studies in India have examined intradistrict and socioeconomic variation in death registration. The recent National Family Health Survey (2019–2021) in India provides data that allow examination of death registration at the district level by socioeconomic characteristics. Thus, we aimed to investigate the differences in death registration in India according to district (administrative units) and socioeconomic characteristics. Such information could help identify specific groups and areas where registration is low that should be prioritized to improve death registration coverage.

## Methods

### Data source

We used data from the Indian DHS, also known as the National Family Health Survey, 2019–2021, conducted under the Ministry of Health and Family Welfare and the International Institute of Population Sciences, Mumbai.^12^ This survey includes a nationally representative sample and gives reliable information on household populations, housing characteristics, fertility, family planning, maternal and child health, stillbirth, infant and child mortality, death registration, nutrition and morbidity for 28 states, eight union territories and 707 districts. In the 2019–2021 survey information was collected from 636 699 Indian households, with a response rate of 98%. In the selected households, 724 115 women and 101 839 men were interviewed. We included a total sample of 84 390 individuals who died in the 3 years before the survey (since 2016) in the final analysis.

### Data collection

The National Family Health Survey, 2019–2021 provides information on the number of deaths in a household since 2016. Family members who experienced the death of a member of their household were also asked if the death had been registered with the civil authority. We created a dependent binary variable for death registration with a civil authority, where 1 indicated the death had been registered, and 0 indicated it had not.

We considered the demographic and socioeconomic variables of the deceased person. We categorized the variables as: age (0–4, 5–14, 15–24, 25–34, 35–49, 50–64, 65–98 years); gender (male, female); place of residence (urban, rural); region (north, north-east, south, central, east, west); highest level of education completed by at least one of the household members (illiterate, primary, secondary, higher); religion of the head of household (Hindu, Muslim, other); caste of the head of household (scheduled caste, scheduled tribe, other backward caste, other); wealth quintile of the household (poorest, poorer, middle, richer, richest); and type of family (nuclear family: married couples living only with their children; non-nuclear family: married couples living with their parents and other family members). We also included welfare variables categorized as: household has a below poverty level card, which allows purchase of some food grains at subsidized costs (yes, no); household has a bank account (yes, no); and the usual members of the household have health insurance (yes, no).

In addition, we considered district level variables such as: proportion of the population that were scheduled tribes; proportion of households where at least one member had completed secondary education; mean household size; proportion of households with a bank account; proportion of households covered by health insurance; proportion of households living in urban areas; and proportion of births taking place in a health-care institution in a district. These variables are indicators of the socioeconomic level of the districts which may affect death registration.

### Data analysis

First, we estimated death registration at the district level. We also estimated death registration by demographic and socioeconomic characteristics of the deceased. We use the *χ*^2^ test to examine the association of demographic, socioeconomic and welfare variables associated with death registration of the deceased person. We then used multilevel binary logistic regression analysis with random intercept and fixed slope to calculate the adjusted odds ratio (aOR) at three levels – level 1: individual; level 2: district; level 3: state – with 95% confidence intervals (CI). We used survey weights to adjust for the design of the study. We considered a *P*-value less than 0.05 to be statistically significant. Multilevel analysis generates variance at each level, providing the technical advantage of assessing unobserved effects at each level.

We used Akaike information criteria and log-likelihood for model comparison. We checked multicollinearity using the variance inflation factor. We examined intraclass correlation to estimate the percentage variance explained at the district and state levels. We used R, version 4.0.2 (R Foundation, Vienna, Austria) for all analyses. We also mapped the proportion of registered deaths by district using the open source geographic information system QGIS software.^13^

## Results

Our sample included 84 390 individuals who died in the 3 years before the survey. Table 1 shows the characteristics of the deceased persons and the households in which they lived. Most deaths (70.8%; 59 748/84 390) in our sample were registered with a civil authority, as reported by the deceased person’s household members.

**Table 1:** Characteristics of the deceased persons and their households, India, 2019–2021

Characteristic^a^	No. of deaths	% of total deaths (SE)	95% CI
**Death registered**			
No	24 642	29.2 (0.2)	28.8–29.6
Yes	59 748	70.8 (0.2)	70.4–71.2
**Age of deceased person, years**			
0–4	4 253	5.0 (0.1)	4.9–5.2
5–14	2 371	2.8 (0.1)	2.7–2.9
15–24	3 302	3.9 (0.1)	3.8–4.0
25–34	3 568	4.2 (0.1)	4.1–4.4
35–49	8 166	9.7 (0.1)	9.5–9.9
50–64	16 847	20.0 (0.1)	19.7–20.2
65–98	45 883	54.4 (0.2)	54.0–54.7
**Gender of deceased person**			
Male	48 323	57.3 (0.2)	56.9–57.6
Female	36 063	42.7 (0.2)	42.4–43.1
**Residence**			
Urban	23 908	28.3 (0.2)	28.0–28.6
Rural	60 482	71.7 (0.2)	71.4–72.0
**Region**			
North	9 616	11.4 (0.1)	11.2–11.6
North-east	2 467	2.9 (0.1)	2.8–3.0
South	18 298	21.7 (0.1)	21.4–22.0
Central	22 411	26.6 (0.2)	26.3–26.9
East	20 618	24.4 (0.1)	24.1–24.7
West	10 981	13.0 (0.1)	12.8–13.2
**Highest education level completed**			
Illiterate	25 572	30.4 (0.2)	30.1–30.7
Primary	15 391	18.3 (0.1)	18.1–18.6
Secondary	35 027	41.7 (0.2)	41.3–42.0
Higher	8 055	9.6 (0.1)	9.4–9.8
**Wealth quintile**			
Poorest	18 630	22.1 (0.1)	21.8–22.4
Poorer	18 242	21.6 (0.1)	21.3–21.9
Middle	17 200	20.4 (0.1)	20.1–20.7
Richer	15 952	18.9 (0.1)	18.6–19.2
Richest	14 367	17.0 (0.1)	16.8–17.3
**Religion**			
Hindu	70 264	83.3 (0.1)	83.0–83.5
Muslim	9 538	11.3 (0.1)	11.1–11.5
Other	4 589	5.4 (0.1)	5.3–5.6
**Caste** ^b^			
Scheduled caste	19 355	24.1 (0.2)	23.8–24.4
Scheduled tribe	7 390	9.2 (0.1)	9.0–9.4
Other backward caste	35 645	44.5 (0.2)	44.1–44.8
Other	17 790	22.2 (0.1)	21.9–22.5
**Family type** ^c^			
Nuclear	42 788	50.7 (0.2)	50.4–51.0
Non-nuclear	41 602	49.3 (0.2)	49.0–49.6
**Household has a below poverty level card**			
No	44 354	52.6 (0.2)	52.3–53.0
Yes	39 911	47.4 (0.2)	47.0–47.7
**Household has a bank account**			
No	3285	3.9 (0.1)	3.8–4.0
Yes	81 085	96.1 (0.1)	96.0–96.2
**Household members have health insurance**			
No	50 278	59.9 (0.2)	59.5–60.2
Yes	33 712	40.1 (0.2)	39.8–40.5

CI: confidence intervals; SE: standard error.

^a^ Weighted sample size: 84 390.

^b^ The caste information was missing for 4210 deaths. We categorized them as “missing” while carrying out the regression analysis.

^c^ Nuclear family: married couples living only with their children; non-nuclear family: married couples living with their parents and other family members.

Death registration for males was higher than for females across the districts. There was also a wide geographical disparity in death registration (available in the online repository).^14^ In our study period, in 122 of 707 districts less than 40% of female deaths were registered while in 251 districts, more than 80% of female deaths were registered. In Bihar, in 37 of 38 districts, less than 60% of female deaths were registered. In all the districts in Jharkhand, 18 of 26 districts in Arunachal Pradesh and 70 of 75 districts in Uttar Pradesh, less than 60% of female deaths were registered. In the Mumbai district of Maharashtra 100% of female deaths were registered while in Kurung Kumey district of Arunachal Pradesh only 5% of deaths in females were registered.

For males, in 53 of the 707 districts, 40% of deaths were registered, whereas in 354 districts more than 80% of deaths were registered (online repository).^14^ In 17 districts, including Mumbai, Kannur, Rajkot, Thrissur, Kancheepuram and Valsad, 100% of male deaths were registered. The lowest proportions of deaths registered for men were in Kurung Kumey (10%) and Upper Subansiri (10%) districts of Arunachal Pradesh. 

All the independent variables examined were significantly associated with death registration (*P* < 0.001 for all; Table 2). Death registration was highest for deceased persons aged 50–64 years (78.1%; 13 152/16 847) and 65–98 years (72.0%; 33 027/45 883) and was lowest for deceased children 0–4 years (34.7%; 1475/4253). Overall, 74.6% (36 039/48 323) of male deaths were registered compared with 65.7% (23 697/36 063) of female deaths. Death registration was higher in: urban areas; households where a member had completed more than a secondary level of education; households with a higher wealth status; nuclear households; and households with a bank account and health insurance (Table 2).

**Table 2:** Death registration by sociodemographic characteristics of the deceased persons and their households, India, 2019–2021

Characteristic	Total deaths, no.	Deaths registered, no. (%)	*P*
**Age of deceased person, years**			< 0.001
0–4	4 253	1 475 (34.7)	
5–14	2 371	958 (40.4)	
15–24	3 302	1 957 (59.3)	
25–34	3 568	2 648 (74.2)	
35–49	8 166	6 521 (79.9)	
50–64	16 847	13 152 (78.1)	
65–98	45 883	33 027 (72.0)	
**Gender of deceased person**			< 0.001
Male	48 323	36 039 (74.6)	
Female	36 063	23 697 (65.7)	
**Residence**			< 0.001
Urban	23 908	19 911 (83.3)	
Rural	60 482	39 827 (65.9)	
**Region**			< 0.001
North	9 616	8 014 (83.3)	
North-east	2 467	1 580 (64.1)	
South	18 298	15 928 (87.1)	
Central	22 411	12 568 (56.1)	
East	20 618	11 649 (56.5)	
West	10 981	9 999 (91.1)	
**Highest education level completed**			< 0.001
Illiterate	25 572	16 018 (62.6)	
Primary	15 391	10 860 (70.6)	
Secondary	35 027	26 029 (74.3)	
Higher	8 055	6 582 (81.7)	
**Wealth quintile**			< 0.001
Poorest	18 630	9 639 (51.7)	
Poorer	18 242	11 746 (64.4)	
Middle	17 200	12 850 (74.7)	
Richer	15 952	12 996 (81.5)	
Richest	14 367	12 506 (87.1)	
**Religion**			< 0.001
Hindu	70 264	49 845 (70.9)	
Muslim	9 538	6 181 (64.8)	
Other	4 589	3 710 (80.8)	
**Caste** ^a^			< 0.001
Scheduled caste	19 355	13 140 (67.9)	
Scheduled tribe	7 390	4 962 (67.1)	
Other backward caste	35 645	24 741 (69.4)	
Other	17 790	13 679 (76.9)	
**Family type** ^b^			< 0.001
Nuclear	42 788	30 153 (70.5)	
Non-nuclear	41 602	29 587 (71.1)	
**Household has a below poverty level card**			< 0.001
No	44 354	32 103 (72.4)	
Yes	39 911	27 547 (69.0)	
**Household has a bank account**			< 0.001
No	3285	2 286 (69.6)	
Yes	81 085	57 441 (70.8)	
**Household members have health insurance**			< 0.001
No	50 278	33 540 (66.7)	
Yes	33 712	25 908 (76.9)	
**Total**	**84 390**	**84 390 (100.0)**	**NA**

NA: not applicable.

^a^ The caste information was missing for 4210 deaths. We categorized them as “missing” while carrying out the regression analysis.

^b^ Nuclear family: married couples living only with their children; non-nuclear family: married couples living with their parents and other family members.

Table 3 shows the result of multilevel binary logistic regression of demographic, socioeconomic and welfare factors. Compared with death registration of children 0–4 years, the likelihood of death registration was higher in all other age groups: 25–34 years (aOR: 4.35; 95% CI: 3.87–4.89); 35–49 years (aOR: 5.05; 95% CI: 4.58–5.57); 50–64 years (aOR: 4.18; 95% CI: 3.86–4.52); and 65–98 years (aOR: 2.92; 95% CI: 2.70–3.15). Female deaths were less likely to be registered than male deaths (aOR: 0.61; 95% CI: 0.59–0.64) as were deaths in rural areas compared with deaths in urban areas (aOR: 0.79; 95% CI: 0.75–0.84). Regionally, deaths were less likely to be registered in the north-east region (aOR: 0.35; 95% CI: 0.17–0.73) and east region (aOR: 0.28; 95% CI: 0.12–0.67) compared with the north region. The west region had a higher likelihood of death registration than the north region (aOR: 2.94; 95% CI: 1.13–7.69). The differences were not significant for south and central regions.

**Table 3:** Factors associated with death registration, India, 2019–2021

Variable	aOR (95% CI)
**Age of deceased person, years**	
0–4	Reference
5–14	1.21 (1.08–1.36)
15–24	2.61 (2.32–2.94)
25–34	4.35 (3.87–4.89)
35–49	5.05 (4.58–5.57)
50–64	4.18 (3.86–4.52)
65–98	2.92 (2.70–3.15)
**Gender of deceased person**	
Male	Reference
Female	0.61 (0.59–0.64)
**Residence**	
Urban	Reference
Rural	0.79 (0.75–0.84)
**Region**	
North	Reference
North-east	0.35 (0.17–0.73)
South	1.46 (0.68–3.14)
Central	0.39 (0.15–1.05)
East	0.28 (0.12–0.67)
West	2.94 (1.13–7.69)
**Highest education level completed**	
Illiterate	Reference
Primary	1.08 (1.02–1.15)
Secondary	1.26 (1.21–1.31)
Higher	1.54 (1.42–1.66)
**Wealth quintile**	
Poorest	Reference
Poorer	1.25 (1.17–1.32)
Middle	1.52 (1.44–1.61)
Richer	1.84 (1.74–1.95)
Richest	2.39 (2.21–2.58)
**Religion**	
Hindu	Reference
Muslim	0.82 (0.76–0.89)
Other	0.97 (0.88–1.07)
**Caste**	
Scheduled caste	Reference
Scheduled tribe	0.85 (0.79–0.89)
Other backward caste	1.05 (0.99–1.11)
Other	1.13 (1.06–1.20)
**Family type** ^a^	
Nuclear	Reference
Non-nuclear	1.02 (0.98–1.06)
**Household has a below poverty level card**	
No	Reference
Yes	1.06 (1.02–1.10)
**District-level demographic variable**	
Proportion of scheduled tribes in a district	0.91 (0.75–1.11)
Proportion of households with secondary education	0.79 (0.54–1.14)
Mean household size	1.01 (0.95–1.07)
**District-level welfare variable**	
Proportion of households with a bank account	2.18 (0.36–13.24)
Proportion of households with health insurance	0.99 (0.83–1.18)
Proportion of households living in urban areas	1.15 (0.68–1.95)
Proportion of households with institutional birth	1.01 (0.72–1.41)

aOR: adjusted odds ratio; CI: confidence intervals.

^a^ Nuclear family: married couples living only with their children; non-nuclear family: married couples living with their parents and other family members.

Note: The final model included 77 697 observations because data were missing for some independent variables.

Higher education of the household members was associated with greater odds of death registration with the greatest odds in households with a member with post-secondary school education (aOR: 1.54; 95% CI: 1.42–1.66). Household wealth status was also significantly associated with death registration with households in the richest quintile most likely to register a death compared with the poorest quintile (aOR: 2.39; 95% CI: 2.21–2.58). Religion and caste were significantly associated with death registration. The odds of death registration were lower in Muslim than Hindu households (aOR: 0.82; 95% CI: 0.76–0.89), and in scheduled tribe households than scheduled caste households (aOR: 0.85; 95% CI: 0.79–0.89). The likelihood of death registration was higher in households with a below poverty level card (aOR: 1.06; 95% CI: 1.02–1.10). We did not find any significant associations between death registration and the district-level variables (Table 3).

We found no collinearity between the independent variables (mean variance inflation factor: 1.43). The random variance value at the state was 0.55 (standard error, SE: 0.74); for district and individual levels this value was 0.16 (SE: 0.40) and 3.29 (SE: 1.81), respectively. In addition, the interclass correlation coefficient value was 0.14 (SE: 0.37) at the state level, indicating that 14.0% of the total variation in death registration was explained by between-state differences while the remaining 86.0% was explained by within-state differences. The interclass correlation coefficient value at the district level was 0.04 (SE: 0.20), indicating that 4% of the total variation in death registration was explained by between-district differences. 

## Discussion

Just under three quarters of deaths were registered at the national level but this figure varied from 5% to 100% at the district level. The wide disparity in death registration is due to unequal socioeconomic development, lack of awareness and lack of health facilities. West regions showed a higher level of death registration than the north regions. Most western states are performing better in terms of the SDGs,^15^ which could result in a higher death registration level. Previous studies also reported a higher death registration level in western states of India.^9^^,16,17^

Our results show a clear gender disparity in death registration with lower levels of registration for females. This difference could be attributed to a lower proportion of women employed in the formal sector and hence a perceived lower need to register female deaths.^18,19^ In addition, globally females tend to have a longer life expectancy than males which is true also in India. This situation may mean that there is no one to register a wife’s death after the death of the husband in a single household.^20^ A higher proportion of accidental deaths among males (which are usually the subject of a police investigation) may lead to higher odds of death registration among males.^21,22^ Previous studies in India have also reported lower death registration among females.^9^^,16^

Death registration was lower among all disadvantaged groups, such as women, rural residents, economically poor groups and deprived caste groups. An earlier study found that socioeconomic development is a main determinant of health-care utilization programmes, strategies and activities, including reporting a death to a civil authority.^23^ Socioeconomic development, such as higher level of education or income, led to achieving other factors related to strengthening death registration services.^23^ We found that deaths of older people were more likely to be registered, which may be associated with inheritance, pension claims and insurance.^22,24^ This finding is in line with a global study.^22^ We also saw low death registration among children, perhaps because of lack of financial benefits from the death of a child and stigma related to premature death of a child, which is similar to the findings of other studies.^17,23,25^

Place of residence of the deceased person was significantly associated with his or her death registration. In rural areas, most adults are employed in informal sectors such as farming, cultivation, construction work and fishing, which seldom provide social security such as a family pension. Lack of motivation, few incentives and poor access to death registration services resulted in lower registration in rural areas as compared with urban areas.^18,26^ Our findings concur with official figures of death registration level in rural and urban areas.^9^ The level of education completed by household members of the deceased was significantly associated with death registration. Previous studies show that knowledge-related barriers limit the extent of death registration in low- and middle-income countries.^23,27^

Death registration was higher in economically well-off households. Our finding concurs with a study that found household income was significantly associated with death registration.^23^ Another study showed that wealthier households take sick household members to hospital and consequently can afford the costs incurred in death registration.^24^

Death registration was lower among Muslim and scheduled tribe households. Cultural beliefs and traditional practices have been reported as reasons for delay in death registration in Indonesia.^28^ In China, most people die at home and death information is not reported to civil authorities.^29^ Underreporting of deaths among Muslims and scheduled tribes in our study may be because of some cultural beliefs and this issue needs further investigation.

Households that had a below poverty level card were more likely to register deaths. A family member who has this card may seek financial assistance if the deceased person died prematurely. To obtain this assistance may require the death to be registered, which may motivate registration. Under national family benefit schemes in India, a lump sum family benefit of 10 000 rupees (about 123 United States dollars) is provided to households in case of death of a primary breadwinner. Only families who hold below poverty level cards are eligible for this scheme.^30^

Our findings suggest that variation in death registration is mostly explained by individual-level variables. Living in a district where a higher proportion of secondary-educated people live would likely result in a high level of awareness of the death registration process. However, most of the district-level variables were not significantly associated with death registration in our study.

While our findings indicate that poorer socioeconomic background was associated with a lower death registration, earlier studies found that contextual factors led to insufficient death registration. For example, a district has several registration offices and the distance to the registration centre can affect death registration.^31,32^ A study in Bihar recently found that people faced challenges in reporting births and deaths because of poor services at the registration centres, higher indirect opportunity costs, such as loss of daily wages and time, and the demand of bribes by the civil registration staff for providing certificates.^25^

More than three quarters of the deaths in India occur at home and most of these deaths do not have a certified cause.^33^ When a death is not registered and a cause is not certified, understanding the risk factors for death at the national, regional and local level becomes a challenge. Without this understanding, it is difficult for policy-makers to intervene and formulate policies related to cause of death at the local level.^34^ Mapping death registration helps to identify vulnerable subgroups and districts lagging behind in registration. This information also helps to raise awareness about the importance of death registration for recording causes of death, which allows interventions to be developed to reduce preventable deaths.

Our study has some limitations. First, death registration data were reported by the respondents; the interviewer did not check for a death certificate. Therefore, our findings may be an overestimate of death registration if, for example, a respondent mistakenly thought that the medical records of the deceased person were the death certificate. Second, part of the data collection was done in the post-COVID-19 period. Because of the government’s compensation scheme for deaths related to COVID-19, more deaths may have been registered than usual. Third, the National Family Health Survey is a cross-sectional survey; therefore, the association between death registration and socioeconomic variables does not indicate causality. Despite these limitations, our findings fill a gap in the existing literature on death registration in India.

Our findings suggest a targeted approach to increase death registration coverage in central, east and north-eastern regions of India. Because death registration is lower in certain groups (females, individuals from deprived castes and less educated people) in almost all regions of India, we recommend strategies to raise awareness of documentation of death for these groups through, for example, mass media and community programmes. Providing financial assistance for funeral rites, education loans to orphans and social security to the deceased person’s family member after reporting the death to a civil authority can help to increase the death registration in India. In addition, the use of modern technology to improve death registration, such as mobile telephone applications for real-time death reporting, will likely increase death reporting.
